# Drug‐induced liver injury associated with selective androgen receptor modulators in an adolescent patient

**DOI:** 10.1002/jpr3.70041

**Published:** 2025-06-10

**Authors:** David J. Katibian, Laura E. Bauman, Katayoon Shayan, D. Brent Polk

**Affiliations:** ^1^ Department of Pediatrics University of California, La Jolla San Diego California USA; ^2^ Division of Gastroenterology, Hepatology and Nutrition Rady Children's Hospital San Diego California USA; ^3^ Department of Pathology University of California, La Jolla San Diego California USA; ^4^ Division of Pathology, Rady Children's Hospital San Diego California USA

**Keywords:** hepatotoxicity, SARMs, supplements

## Abstract

Selective androgenic receptor modulators (SARMs) have similar properties to anabolic steroids but bind to androgen receptors in a tissue‐specific manner. Studies have investigated the benefits of SARMs in promoting bone and muscle growth while limiting the adverse effects of androgenic stimulation of other organs. However, an increase in the reported cases of hepatotoxicity in adults secondary to SARMs found in bodybuilding supplements has raised concerns about their safety. We report the first known adolescent case of SARMs‐associated liver injury with a primarily hepatocellular pattern of injury. Previous reports in adults had shown a primarily cholestatic pattern of injury. Our case highlights, the variations in the phenotypic and histologic patterns of injury based on the amount and type of SARMs. The purpose of this case report is to shed light on the potential spectrum of liver injury related to SARMs use and continue to raise awareness of the associated health risks.

## 1 INTRODUCTION

Elevated transaminases and hepatitis result from varied etiologies. Selective androgen receptor modulators (SARMs) are therapeutic compounds with similar properties to anabolic steroids but with reduced androgenic properties. SARMs reportedly selectively bind androgenic receptors in certain tissue including muscle and bone, and have been used investigationally for diseases like osteoporosis and cachexia.^1^ They are also popular for performance enhancement. In 2017, the Food and Drug Administration (FDA) warned against supplements containing SARMs because of life‐threatening reactions including liver toxicity.^2^ The majority of case reports describing liver injury in patients exposed to SARMs^3^, ^4^, ^5^, ^6^, ^7^, ^8^, ^9^ are in adult patients with cholestatic patterns of injury. To our knowledge, this is the first documented case of SARMs‐induced liver injury in an adolescent patient with a primarily hepatocellular pattern of injury.

## 2 CASE REPORT

A 17‐year‐old male patient with no significant medical history presented to the emergency department with 2 weeks of fatigue, headaches, nausea, and generalized abdominal pain. Laboratory evaluation revealed hepatitis with preserved synthetic function. He denied history of elevated serum transaminases or pertinent family history. He recently traveled to Tecate (Mexico) and denied sick contacts, consumption of undercooked foods, alcohol, or acetaminophen ingestion. Immunizations were current except for hepatitis A and B series. He had been taking a daily Loratadine but denied any other medication use. He reported taking “Alphaphorm Halo‐T,” an over‐the‐counter muscle‐building supplement containing SARMs, daily for 1 month before presentation. He denied fevers, congestion, emesis, diarrhea, hematochezia, rashes, acholic stools, or arthralgias.

Vital signs were within normal limits and physical examination was revealing only for scleral icterus without cutaneous jaundice. His initial biochemical evaluation included alanine aminotransferase (ALT) 1224 U/L (5‐52 U/L), aspartate aminotransferase (AST) 552 U/L (10‐45 U/L), total bilirubin (TB) 1.2 mg/dL (0.1–1 mg/dL), direct bilirubin (DB) < 0.1 (0.0–0.3 mg/dL), and gamma‐glutamyl transferase (GGT) 201 U/L (11–34 U/L). Other initial biochemical evaluation included a normal complete blood count, serum electrolytes, glucose, albumin, alkaline phosphatase, creatine kinase, and prothrombin time (PT)/international normalized ratio (INR). Additional evaluations for autoimmune hepatitis, Wilson's disease, alpha‐1‐antitrypsin deficiency, and viral hepatitis (including hepatitis E) were unremarkable. Iron panel showed elevated iron 205 µg/dL (30–120 µg/dL), total iron‐binding capacity (TIBC) 532 µg/dL (250–400 µg/dL), and ferritin 423 ng/mL (6–70 ng/mL) but normal iron saturation 39% (20%–50%). Abdominal ultrasound had no abnormality and a normal doppler evaluation.

**Figure 1:**
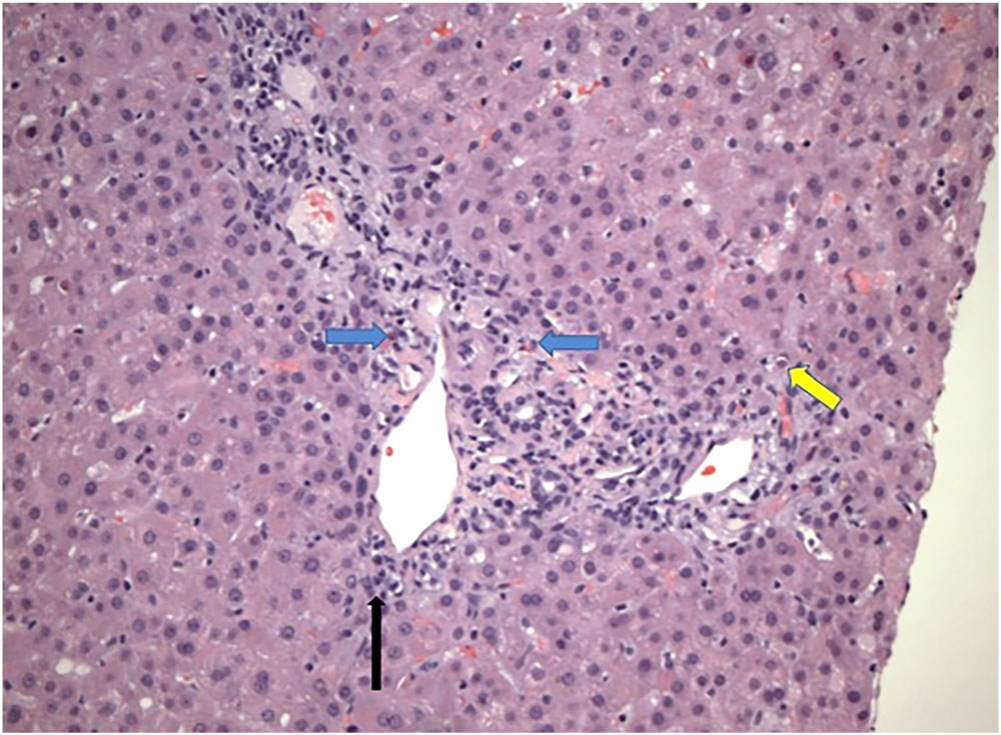
Liver biopsy specimen: H&E stain showing mixed. portal tract inflammation expanded by increased numbers of lymphocytes, neutrophils. and eosinophils (blue arrows). Necrotic hepatocytes (yellow arrow) and interface. Hepatitis (black arrow) are also noted. H&E, hematoxylin and eosin.

**Figure 2:**
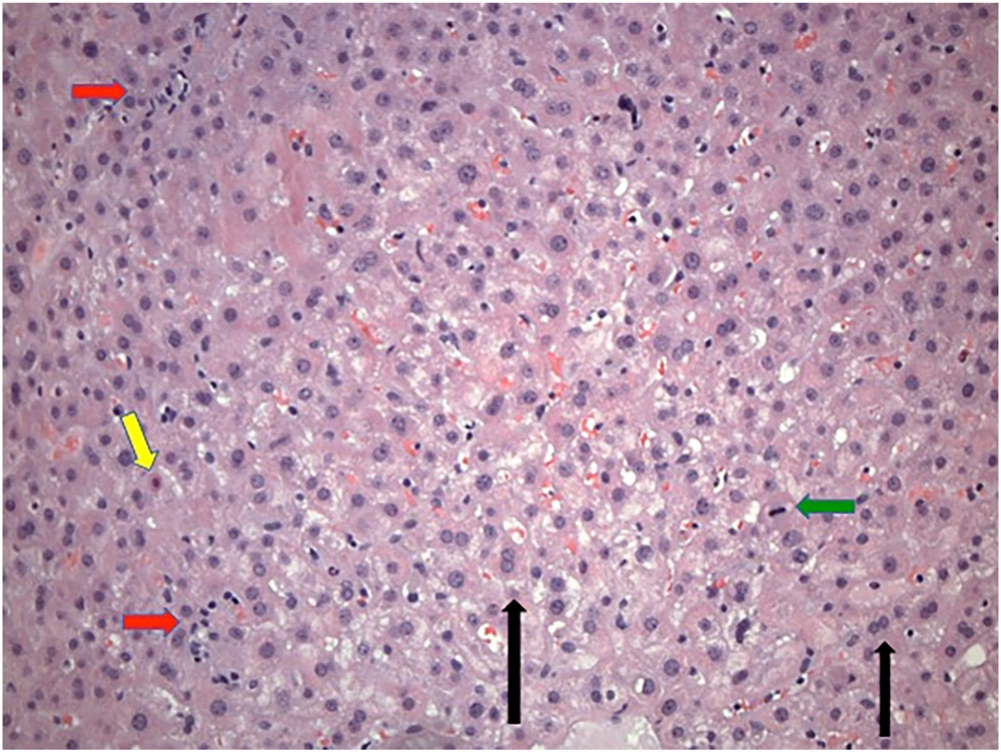
Liver biopsy specimen: H&E stain showing lobular inflammation (red arrows) with binucleated and multinucleated hepatocytes (black arrows), mitotic figures (green arrow), and necrotic hepatocyte (yellow arrow). H&E, hematoxylin and eosin.

Serum markers of hepatitis peaked on hospital day four (ALT 1662 U/L [5‐52 U/L], AST 906 U/L [10–45 U/L], TB 1.6 mg/dL [0.1–1 mg/dL] (DB < 0.1 mg/dL), and GGT 228 U/L [11–34 U/L]). Percutaneous liver biopsy at that time revealed a primarily hepatocellular pattern of injury with portal and lobular mixed inflammation. Portal triads were mildly expanded by lymphocytes, neutrophils, and eosinophils with rare plasma cells (Figure 1, see Supplemental Digital Content S1B). Hepatic lobules had bi‐nucleated and multinucleated hepatocytes with rare foci of hepatocellular cholestasis. Multiple necrotic hepatocytes, mitotic figures, and focal interface hepatitis were present (Figure 2). Trichrome stain showed mild fibrous expansion of portal triads (see Supplemental Digital Content S1C). There was no evidence of cholangitis, vasculitis, viral inclusions or significant steatosis. Iron staining was negative; quantitative iron from biopsy was low at 146 µg/g (400–2200). Immunohistochemistry for cytomegalovirus and Epstein‐Barr virus were negative.

**Figure 3:**
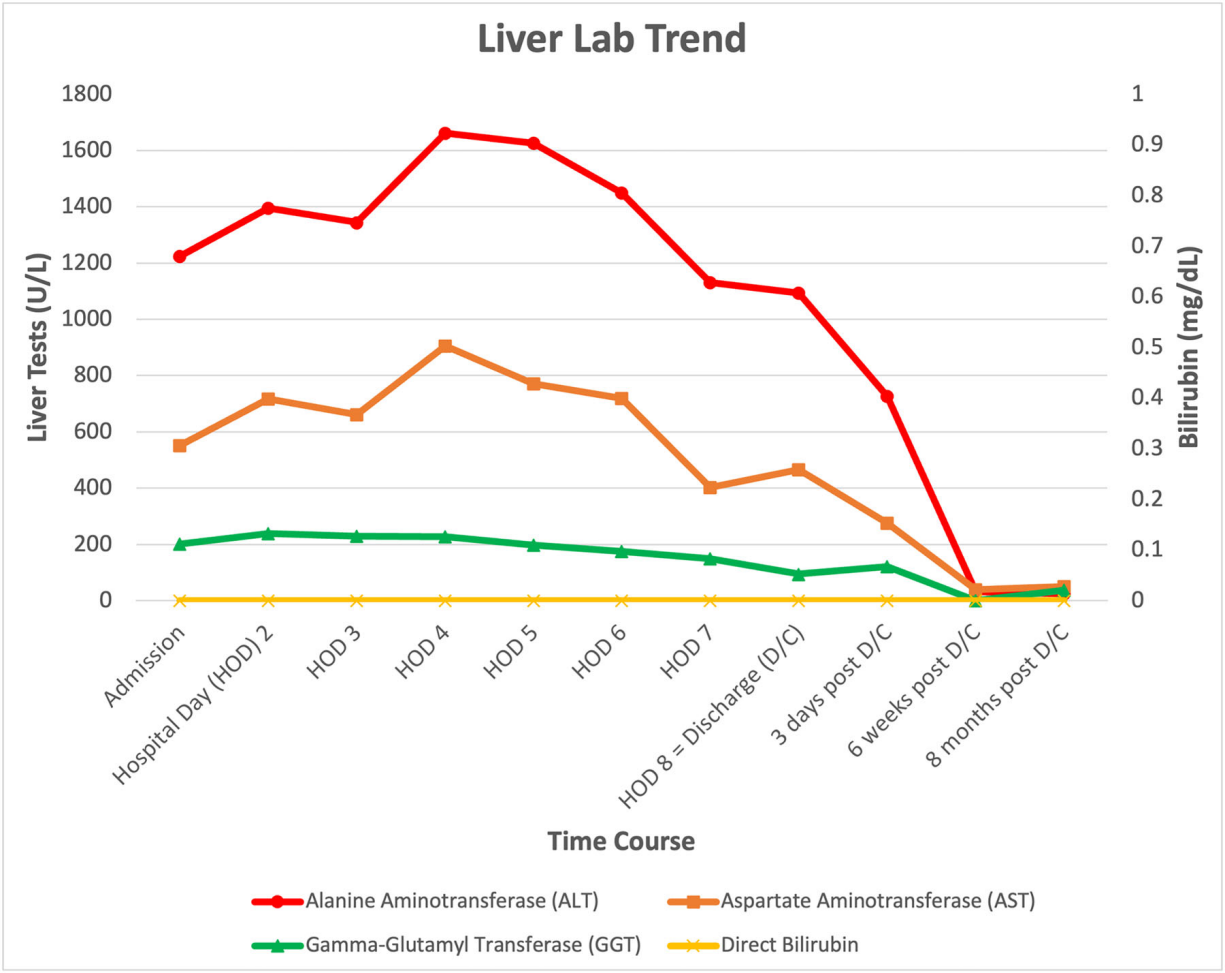
Liver lab trend.

A presumptive diagnosis of drug‐induced liver injury (DILI) was made secondary to use of SARMs. Symptoms improved and serum transaminases downtrended at discharge on hospital day eight (ALT 1093 U/L [5–52 U/L], AST 465 U/L [10–45 U/L]). Repeated serologic evaluation a few days later demonstrated continued improvement, with eventual normalization 2 months after discontinuation of the SARMs (Figure 3). Synthetic function as evidenced by PT/INR remained normal throughout the hospital course.

## 3 DISCUSSION

As SARMs become more popular and accessible, there has been an increase in SARMs‐associated liver injuries in adults.^3^, ^4^, ^5^, ^6^, ^7^, ^8^, ^9^ To our knowledge, this is the first reported case of SARMs‐associated liver injury in an adolescent patient, with notable phenotypic and histologic differences. In most adult cases, patients had significant conjugated hyperbilirubinemia and/or histologic evidence of a mixed or cholestatic pattern of liver injury with minimal inflammation. In contrast, we observed significant serologic hepatitis, the absence of biochemical or histologic evidence for cholestasis, and a predominantly hepatocellular pattern of injury on histologic evaluation. Weinblatt et al reported a case of a 31‐year‐old male who presented with a primarily hepatocellular pattern of injury after taking Enobosarm for 3 weeks. Total bilirubin was normal without evidence of cholestasis although he did not undergo a liver biopsy.^9^ The degree of hepatitis in this adult patient was not as severe (ALT 346 U/L, AST 110 U/L) compared with our case (ALT 1662 U/L, AST 906 U/L). Interestingly, most other reported cases of adult SARMs‐associated liver injury showed only mild‐moderate transaminase elevations to the low hundreds.^5^, ^6^, ^7^, ^8^ As SARMs are unregulated products, they may contain unlisted compounds that could have contributed to more significant hepatotoxicity in our case.

To date, there is no consensus on a pathognomonic pattern of hepatic injury secondary to SARMs. Some of the variation is likely linked to the variation in compounds in different supplements. In our case, the hospital medical team confirmed the “Alphaphorm Halo‐T” supplement contained several different SARMs from a patient‐provided image of the bottle. Upon writing this report, we were unable to locate the supplement to verify ingredients. Due to the unregulated nature of these supplements, we suspect they quickly move through a competitive and lucrative market. Depending on the dose, duration and subtype of SARMs ingested there might be variations in the mechanism of injury and thus the clinical and histologic phenotype. In this case, the binucleated/multinucleated hepatocytes observed on histology were suggestive of accelerated liver cell regeneration after injury. Among all previous published cases, the liver injury was reversible with cessation of the supplement and none required a liver transplant. Patients had reported exposures over a range of 2–12 weeks.

## 4 CONCLUSION

Our case further expands the potential spectrum of liver injury patterns associated with DILI due to SARMs ingestion. After ruling out other etiologies, the diagnosis was made based on the timing of use, improvement upon discontinuation, and histopathologic findings. Our case adds to growing evidence that there may be health risks associated with SARMs supplements. There is an urgent need for higher levels of regulation, especially in vulnerable adolescent patients.

## CONFLICT OF INTEREST STATEMENT

The authors declares no conflicts of interest.

## ETHICS STATEMENT

Informed patient consent was obtained for publication of the case details. This case report did not require IRB or ethics committee approval because it is a medical/educational activity that does not meet the DHHS definition of research and does not contain unique information.

## Supporting information


**Supplemental Digital Content 1B:** H&E stain (40x magnification) with better visualization of eosinophils (blue arrows), necrotic hepatocytes (yellow arrow) and binucleated cells (black arrow).

**Supplemental Digital Content 1C:** Trichrome stain with mild fibrous expansion of portal triads.
