# Supplementary figures and images for: Drug‐induced liver injury associated with selective androgen receptor modulators in an adolescent patient
Source: JPGN Rep. 2025 Jun 10;6(4):515–8. doi: 10.1002/jpr3.70041 (PMC12611585; doi:10.1002/jpr3.70041)

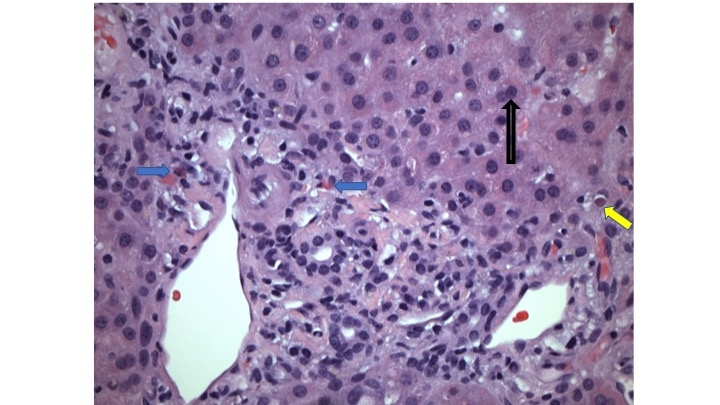

Supplement: Supplementary file 1 — Supplemental Digital Content 1B: H&E stain (40x magnification) with better visualization of eosinophils (blue arrows), necrotic hepatocytes (yellow arrow) and binucleated cells (black arrow). [file JPR3-6-515-s002.tiff]

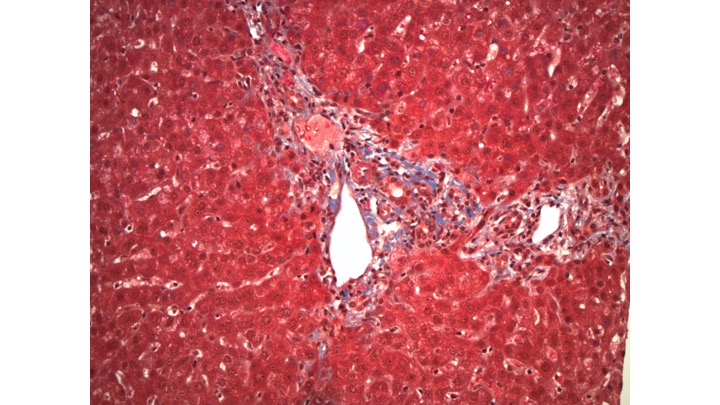

Supplement: Supplementary file 2 — Supplemental Digital Content 1C: Trichrome stain with mild fibrous expansion of portal triads. [file JPR3-6-515-s001.tiff]
